# Provincial division of economic zones based on the improved urban gravity model: A case study of Hunan Province, China

**DOI:** 10.1371/journal.pone.0261205

**Published:** 2021-12-22

**Authors:** Yuanyuan Guo, Shengjie Xi, Mingdou Zhang

**Affiliations:** 1 Department of Urban and Rural Planning, School of Architecture, Tianjin University, Tianjin, China; 2 School of Economics, Dongbei University of Finance and Economics, Dalian, China; Gebze Teknik Universitesi, TURKEY

## Abstract

Regional collaboration and the division of economic zones have been widely discussed for sustainable development. This paper aims at building a framework for analyzing the provincial division of economic zone (DEZ) via considering spatial interaction among regions based on the improved gravity model and clustering approaches. The proposed framework of province DEZ is applied in the case study of Hunan Province, China. The results reveal that Chenzhou city in the south of Hunan Province is always excluded from the DEZ due to its larger external gravity from other cities in neighboring provinces. It also shows that the city components of economic zones (EZs) have experienced a fluctuation at a higher degree from 2002 to 2006 to 2009 while it reached to a stable phase in 2013. Furthermore, cross-provincial regional integration and the highway construction have a significant impact on the change of city components of EZ. The findings are of great potential in regional planning that should be incorporated to the toolkit of regional policy and sustainable development for local governments.

## Introduction

The division of economic zones (DEZ) is a strategy to divide the territory into several distinct sections, namely, economic zones (EZ), according to the law of social labor division. EZs are usually distinctive with each other in resource endowment, economic development level, and development goal, whereas the regions within a specific EZ share similar characteristics of economic development and have strong connections in economy, culture, and society [1]. Based on the theories of economic geography, DEZ is an important basis for rational distribution of productive forces and regional economic cooperation [2].

Over the past several decades, China has generated tremendous economic development with an annual growth rate at 9.9%, becoming the world’s second largest economy in 2010 [3]. However, regional disparity of economic development in China, which is widely believed to be derived from preferential policy, marketization, and globalization [4, 5], has been constantly observed during the process. Additionally, local governments usually prefer to pursue greater economic achievement and compete against each other because the officials’ promotion was obviously GDP performance-oriented [6–8]. Thus, regional cooperation was rarely observed. As a response to the issue of regional inequality and the lack of regional cooperation, DEZ is one of the feasible approaches to promoting the regional development through identifying the difference among regions and enhancing the economic development with differential policies, followed by proposing the overall development goals of regional collaborations [9]. For example, the central government proposed the four large-scale economic zones in 2000s, which divided the whole country into four distinct economic parts, namely, the Northeast, the Northern, the Eastern, and the Central China [10]. The above-mentioned DEZ at national scale has been always viewed as the fundamental basis of national development strategies, such as *Eleventh Five-Year Plan* and subsequent five-year plans [11]. In recent years, a variety of regional division schemes have been proposed for enhancing the economic development from large scale at national level to a minor scale at provincial [12–14], particularly for guiding economic collaboration among different cities [15].

However, both national and provincial schemes of DEZ intends to conduct DEZ via a series of economic thresholds, following by dividing regions into different categories. Therefore, these DEZ schemes usually overemphasized the economic links among regions but lacking of social, cultural, transport, and other types of links [16]. Additionally, the process of provincial DEZ typically focuses on the connections among cities within the administrative boundary of a province, whereas ignoring the external connections from neighboring cities in other provinces [17]. Under the background of regional integration, the barrier from administrative boundary become weak and the regional development tend to encourage the cooperation cross provinces [18, 19], particularly for those cities sharing the distinctive province boundaries.

To address these concerns, this paper aims at building an improved framework for analyzing provincial DEZ. The framework firstly establishes an index system composing of economic and cultural characteristics, followed by improving the gravity model by using the index system to amend the parameters of gravity mass (G) and the gravitational constant (K) as well as applying multiple transport modes to measure the parameter of distance (D). The improved gravity model is then applied in the case study of Hunan Province, using the data from 2002, 2006, 2009, and 2013. The external gravity attractions from other adjacent cities of provinces have been also measured to identify the appropriate components of cities that should be considered into the process of DEZ in Hunan Province.

The rest of this paper is organized as follows. Next section reviews the relevant literature on DEZ in China and other countries, followed by the review on application of gravity model. In the third part, the research design and the amended framework of provincial DEZ is illustrated. Then, the fourth section introduced study area, data and methodology applied. Findings and discussions are presented in the next part, followed by conclusions in the final part.

## Literature review

### Division of economic zone

The economic regional differentiation theory is derived from the classical location theory. According to this theory, DEZ can contribute to revealing the development conditions and the characteristics of economic operation, to objectively analyzing the problems of regional economy, and to adapting the future direction of regional economic development [20]. Additionally, DEZ also helps the government to make overall planning, formulate macro-control policies and promote regional economic growth and regional coordinated development [21, 22]. Practices of DEZ are worldwide, and hence are reviewed and summarized as follow.

DEZ has always been an important research subject in China in past sixty years and is generally conducted in two hierarchies, namely, national DEZ and provincial DEZ [23]. Under the planned economy or socialist market economy, EZ and DEZ bear rich practical experience in Chinese context. In 1950s, China has undergone a weak economy and the resource allocation had to prioritize the regions from coastal to inland. In order to make full utilization of regional advantages among different regions, the central government firstly proposed a nationwide division of six large-scale economic zones (i.e., the Northeast, the Northern, the Eastern, the Central, the Northwest and Southwest China) [24]. Simultaneously, multiple channels for regional collaboration have been established. The scheme of six large-scale zones was the first implementation of DEZ at national level, and was then changed into four-large economic zones, namely, the Northeast, the Northern, the Eastern, and the Central China [10, 11]. Particularly, several national strategies targeted for these four economic zones, such as the *Coastal development First*, the *Rise of Central China*, the *China Western Development*, and the *Revitalization of Northeast China*, have been successively proposed to enhance the regional development within each EZ since the reform and opening up in the 1980s [25–27]. Provinces also tailored its own DEZ depending on the local social-economic condition, geographic resources, and other local characteristics [12–14, 28]. In general, these studies focus on the specific scheme of DEZ and try to establish universal criterion of DEZ [16, 29]. Furthermore, recent studies started to reveal the conflict between EZ and administrative region and to explore the relationship between DEZ and current regional planning [30–32]. For example, the *Major Function Oriented Zoning*, one of the recent implemented national DEZ scheme around 2010s in China, classifies the whole country into four categories according to the resources and environmental carrying capacity and development potential [33]. These zones include priority development zone, key development zone, restricted development zone, and prohibited development zone. The boundary of these zones is no longer consistent with that of province boundary. Additionally, scholars tend to pay more attention to the regional spatial structure of provincial space, on which the provincial DEZ always relies. In practice, local governments often take the regional spatial structure as an important reference for DEZ, and then formulate regional economic strategy in different EZs to promote the whole development of the provincial economy [12, 28]. For example, Duan et al. applied gravity model to quantify the spatial interaction among cities in Jiangsu Province, China, and used 0–1 programming model to divide cities into several distinct economic zones [12]. Li et al. measured the spatial network among cities in Hunan Province, China, by using traffic flow information, and formulated the optimized spatial structure, which contributes to the DEZ of Hunan Province [28].

Scholars in other countries also have implemented different DEZ strategies to promote the regional economic development. Kolovos Chomsky, an economic geographer in the former Soviet Union, defined the “Territorial Productive Complex (TPC)” as a similar concept of economic zone, and the government created a regionalization scheme that included eighteen basic zones in 1961 [34]. To achieve the economic agglomeration effect, some countries in Europe and America also tried to divide regions into several categories on a national scale based on economic development level [35, 36]. The United States, in the 1950s, started an economic development system with four levels based on geographic hierarchy, namely Economic Province, Economic Region, Economic Sub-regions, and State Economic Region [37]. Similarly, the study in Britain recommended that the urban areas should be divided into four categories [38]. The French government has established an economic region system composed of 9 provincial administrative areas, which includes nine cities or urban groups [39, 40]. At provincial level, New York created 10 Regional Councils to develop long-term strategic plans for economic growth [41]. In Alberta, Canada, the local government established Regional Economic Development Alliances (REDAs) to cooperate with different regions in this province [42].

### Gravity model

The gravity model is a hypothesis, which is based on classical Newton’s universal gravitation formula, for interpreting and forecasting the interactions among economic, social, and political fields from a geographical perspective [43, 44]. Tinbergen [45] established a classic form of the gravity model by making an analogy with Newton’s law of gravitation [46]. A milestone was achieved by Anderson in 1979 in the research on a theoretical foundation of gravity modeling [45]. He then described the theoretical development of gravity modeling from traditional to structural gravity models. Their work published in 2003 and contributed to this research community by proposing a term of multilateral resistance in the gravity equation [47]. At the same time, the law of universal gravitation was introduced into sociological research. The sociologists built a subject of "social physics" and used the “gravity model” to identify the social connections among different regions [48]. In these studies, the population size that was usually viewed as “mass” and the distance among the regions were usually used in the gravity model [47, 49–51]. Additionally, some variables such as price, the per capita income and exchange rate were also collectively treated as the virtual “mass” to make it better reflect the actual situation of interaction [12–14, 52, 53].

The gravity model also provides a feasible approach for the analysis of spatial interactions and the trade flows. The structural gravity equation is one step towards a more comprehensive, holistic, and context-sensitive analysis of spatial interaction [54]. It also enriches a broad field in international economics, including the migration modeling [55], foreign direct investment [56], and trade flows [57]. The model has been widely used in other fields, such as commodity, mass communication, international tourism flows or traffic flows, residence-workplace trips, and market area boundaries [58–61].

As one of the major spatial interaction models, gravity model has been gradually applied in calculating the spatial interaction between cities, such as the analysis of urban spatial structure, economic ties between cities, and city`s influence sphere [62]. According to the understanding of spatial interaction, some scholars maintain that different cities will interact with each other and then form an integrated region on a certain spatial scale [63, 64]. Actually, this integrated region is just about the nature of EZ because both of them have the same objective towards regional collaboration. Thus, a provincial DEZ can be fundamentally derived from the analysis of spatial interaction (or gravity model) among the cities within the province. In this case, it endows DEZ with the geospatial property, instead of purely considering the economic property.

### Analytic framework

**Fig 1:**
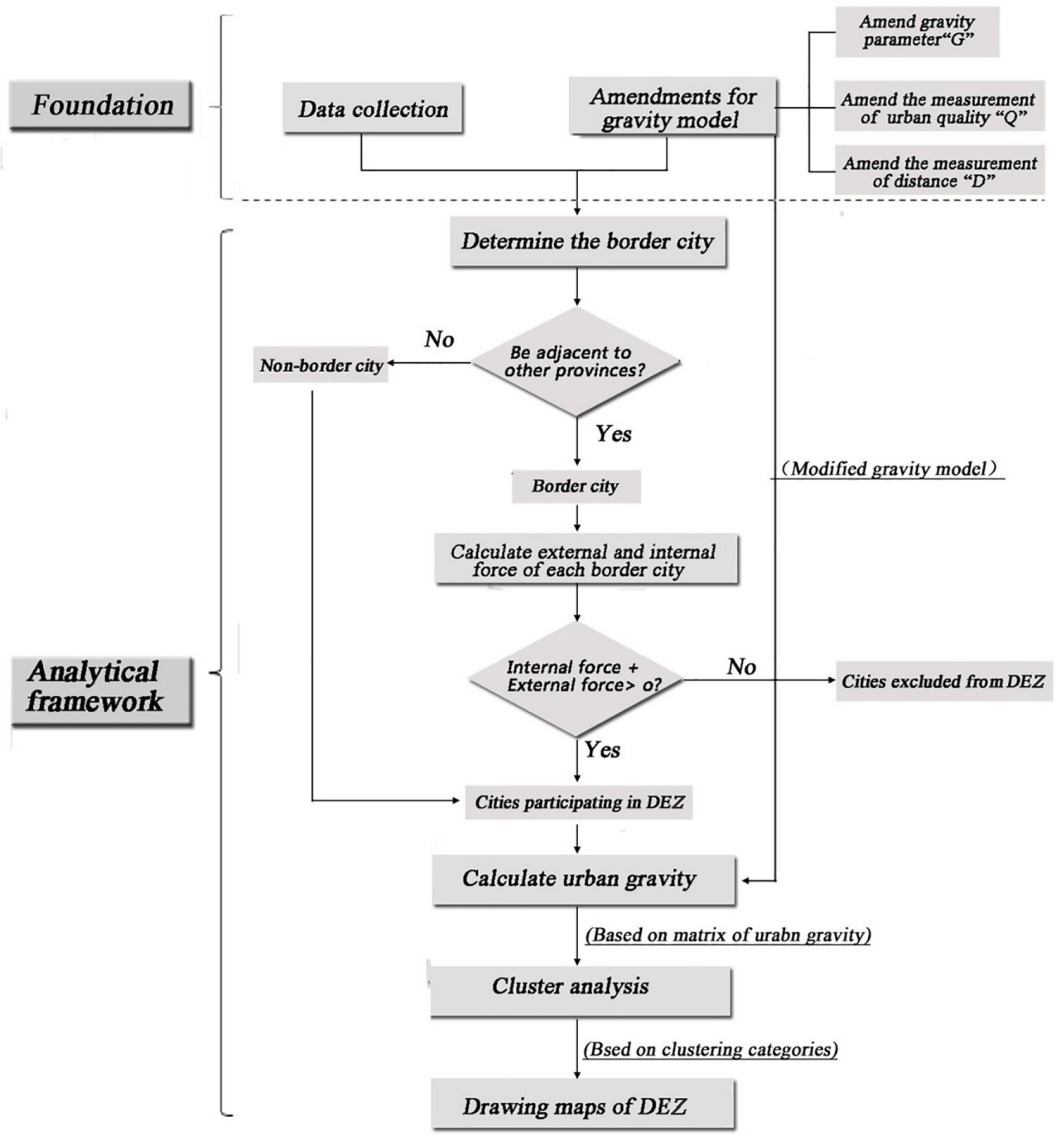
Flow chart of the analytical framework.

Taking Hunan Province as a case study, this paper builds an analytic framework for provincial DEZ (Fig 1). Before applying such a framework to conduct provincial DEZ, this study first modifies the gravity model by proposing the concept of “attractive inertial exponent” to replace the gravitational constant (G) and building up an index system to calculate urban quality and “attractive inertial exponent”. Based on this modified gravity model, the internal and external force, and gravity between different cities are calculated.

This analytical framework for provincial DEZ has the following four steps:

(1) Determine the non-border city and the border city in Hunan Province. The cities in Hunan Province are divided into two categories: non-border city and border city. The non-border city represents the city that shares no boundary with other provinces, while the border city does. The non-border cities are affected by the internal force from the adjacent cities inside of the Hunan Province. The border cities are not only affected by the internal force but also affected by the external force, which results from the adjacent cities in the neighboring provinces.

(2) Calculate internal force and external force of each border city respectively. The modified gravity model is used to calculate each border city`s internal force (FINT) which comes from other cities in Hunan Province, and to calculate the external force (FEXT) which comes from other cities in the neighboring provinces. The direction of these two forces should be opposed. Thus, it is assumed that the direction of the internal force is positive and that of the external is negative. In fact, the magnitude of the internal force or external force is numeric equivalent to the urban gravity between the border city and its adjacent cities. By calculating the sum of the internal and external gravity forces that a border city faces, we can identify it as the border city that should participate the DEZ process if the sum is positive.

(3) Calculating urban gravity among different cities in Hunan Province. For the cities participating in the process of DEZ, the modified gravity model is continually used to calculate the gravity among them. Finally, a matrix of urban gravity is obtained. The matrix can reveal the urban gravity between any two cities in Hunan Province.

(4) Cluster analysis for DEZ based on the matrix of urban gravity. The cluster analysis for DEZ is carried out based on the matrix of urban gravity. Each cluster represents a certain EZ respectively. Then, the spatial distribution of these EZs is presented.

In order to make a clear comparison between the proposed analytical framework of provincial DEZ and the traditional method of DEZ,

This study also used GDP economic data purely, which was traditionally adopted, to achieve the provincial DEZ. Such result of DEZ scheme was set as control group to compare with the provincial DEZ, a new DZE scheme that is clustered from the matrix of urban gravity in this study.

### Study area, data, and methodology

#### Study area

**Fig 2:**
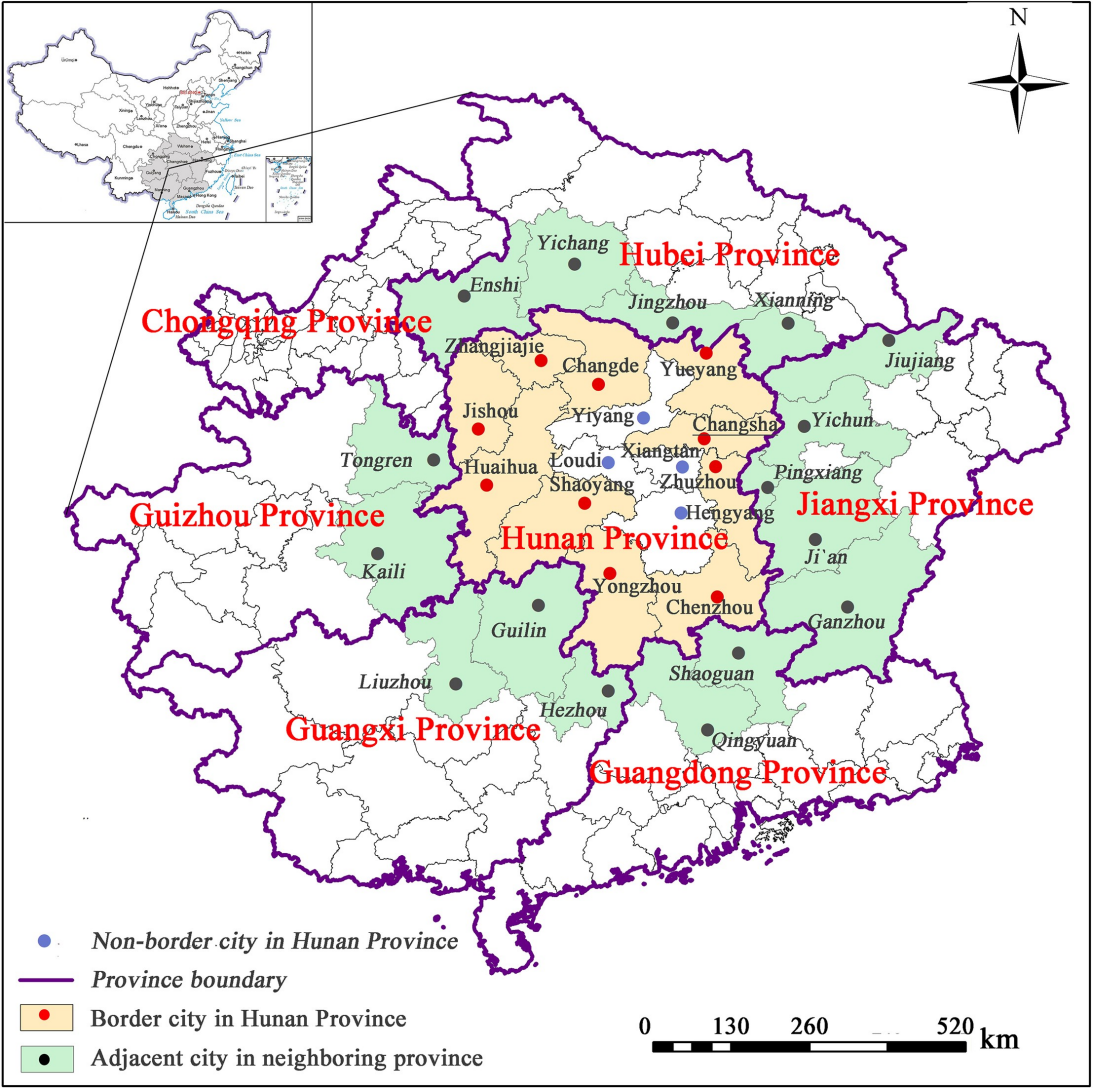
Distribution of cities in Hunan Province and its adjacent cities. (Reprinted from http://www.ngcc.cn/ngcc/ under a CC BY license, with permission from the *National Geomatics Center of China*, original copyright 2012).

As the national strategy of “The Rise of Central China” spread over the years, Hunan Province has witnessed new development opportunities. The average GDP growth from 2009 to 2019 was 9.83%. In 2020, the GDP of Hunan Province reached to 417.81 billion RMB, ranking the 9^th^ in China, whereas the GDP per capital was 60,391 RMB, ranking 14^th^ in China. Hunan Province also has a large population of 69.18 million, which was unevenly distributed in 14 cities in total (Fig 2).

Over the past few decades, the northern region of Hunan Province gradually became the main crop production base because of the excellent natural conditions of the Dongting Lake Plain. The central and southern regions have established a relatively complete industrial portfolio with rich mineral resources and original heavy industry base. The economic development of the western region has been relatively slower than other regions due to its limitations of the natural and geographical resources. In the last ten years, the “Chang-Zhu-Tan Area” has experienced rapid economic development as the result of national policies. In a summary, regional economic imbalance exists in Hunan Province, namely, under-developed in west and prosperity in the east, industry-oriented in the south and agriculture-oriented in the north.

#### Data

National Bureau of Statistics (NBS) of the People`s Republic of China is the basic data source in this study. All of the economic, social and cultural data at four time points (i.e., 2002, 2006, 2009, and 2013) were collected from the China City Statistical yearbook in 2003, 2007, 2010, and 2014 yearbook. Here, it should be noted that the yearbook usually records the data information in the last year, instead of the same year. Also, the National Economic & Social Development Statistics Bulletin, which is linked to the website of NBS, is another major data source to supplement the dataset.

Data for calculating transportation distance were obtained through Bus Ticket Query Network and the Railway Information Network. However, one of the concerns about the data is that the ticket price is dynamic and no system can record this fluctuation. Thus, current price is used to replace the monetary cost at four time points.

### Modified gravity model

As one of the spatial analysis approaches, the gravity model can be used to calculate spatial interaction. However, it cannot be directly deployed into provincial DEZ. To make the model better reveal the spatial interaction between cities, the traditional gravity model needs to be modified. The traditional gravity model and the modified one are shown as the following equations specifically:

$$F_{ij}=G\frac{Q_{i}Q_{j}}{r_{ij}^{b}}$$ (1)

$$F_{ij}=\frac{1}{K_{i}}\frac{{Q_{i}Q}_{j}}{\left(\sum_{m=1}^{n}\lambda_{ij-m}c_{ij-m}T_{ij-m}\right)}$$ (2)

Comparing with the traditional gravity model, the modified gravity model has the following three amendments.

#### Amending gravity parameter *G* based on the inertia theorem

In this paper, the amendment for *G* is inspired by the inertia theorem. Additionally, *G* is replaced by *K* (“attractive inertia exponent”), which is a similar concept with *G* in the gravity model. The magnitude of *K* varies according to different stressed cities (the exact definition will be discussed in detail below). At the same time, it is assumed that the urban gravity between cities is inversely proportional to the stressed city’s “attractive inertia exponent”.

Here, we define the force city as the city that actively pulls other cities with its gravity, and define the stressed city as the city that is attracted by the force city. In fact, each city can be viewed as either a stressed city or a force city. To better illustrate the impact of inertia on the urban gravity between cities, it takes the gravity that city A acts on city B as an example, that is to say, city A is the force city and city B is the stressed city. First, the magnitude of the gravity field that city A and B produce is different, because they have different attributes. In other words, the urban gravity from city A to city B is not the same as that from city B to city A. Second, if the stressed city has higher life quality in terms of the social status, standard of living, infrastructure, and natural environment than the force city does, namely, the stressed city has a larger “attractive inertia exponent”, the degree of influence from the force city to the stressed city will be smaller, because inhabitants in the stressed city will not be easily attracted by the force city. To address the issue, the inertia theorem is applied for the amendment of the gravity parameter *G*.

Multiple Attribute Decision Making (MADM) method was used to calculate *K*. First, an index system (Table 1) is established, followed by determining the weight of each index. Then, the weighted sum method is used to calculate the “attractive inertia exponent” (*K*) value of each city.

**Table 1:** Urban gravity index system.

Classification	The rule layer index	The index layer	Unit
Urban quality	Population	Total population	Ten thousand
	Economy	GDP	Ten million RMB
		Proportion of non-agricultural industries	%
		Total retail sales of social consumer goods	Ten million RMB
	Investment	Investment in fixed assets	Ten million RMB
	Fiscal	Local finance general budget revenue	Ten million RMB
	Finance	Deposit balance of financial institutions	Ten million RMB
	Education	College and university student number	/
	Nature environment	Tourism revenue	Ten million RMB
	Transportation	Passenger traffic volume	Ten thousand
		Cargo volume	Ten thousand ton
	Scale	Built urban area	km^2^
Attract inertia exponent	Social conditions	Unemployment rate	%
	Living standard	Annual income of urban residents	RMB
	Infrastructure	Urban per-capita green area	m^2^
		Urban per-capita road area	m^2^
	Telecommunication	Mobile phone per-ten thousand people	/
		Telecommunication pe capita	/
		Internet household per-ten thousand people	/
	Medical	Hospital bed per- ten thousand people	/
		Health doctor per-ten thousand people	/
Transportation distance	Railway	Rail travel time	min
		Railway transport costs	RMB
	Highway	Highway travel time	min
		Highway transportation costs	RMB

The data pre-processing of these indexes is as follow:

(1) Index selection. The index system for “attractive inertia exponent” covers some indexes that impact urban residents’ life quality. These indexes can be divided into two categories, positive index, and negative index.

(2) Data standardization. Because the data units in the index system are not the same, the data must be standardized. Here, we assume *A* as a set of cities, *A = (A1*, *A2*, *A3 ……An)*; and assume *G* as a set of indexes, *G = (G1*, *G2*, *G3 ……Gm)*. Thus, in terms of the city *Ai*, its index *G*_*j*_ can be noted as *y*_*ij*_ *(i = 1*,*2*,*3……*,*n; j = 1*,*2*,*3……*,*m)*, then *Y = (y*_*ij*_*)*_*n × m*_ represent a matrix related with index set and city set, this matrix can also be called “decision matrix”. The standardization formulas are shown as the following equations:

Positive index standardization:

$$Y_{ij}=(y_{ij}-y_{j-min})/(y_{j-max}-y_{j-min}),\;i=1,2,3\ldots \ldots n;\;j=1,2,3\ldots \ldots \ldots m$$ (3)

Negative index standardization:

$$Y_{ij}=\left(y_{j-max}-y_{ij}\right)/(y_{j-max}-y_{j-min}),\;i=1,2,3\ldots \ldots n;\;j=1,2,3\ldots \ldots m$$ (4)

Where *y*_*j−min*_ refers to the minimum value of the index *G*_*j*_, *y*_*j−max*_ refers to the maximum value of the index *G*_*j*_, *y*_*ij*_ refers to the value of the index *G*_*j*_ of the city *Ai*, *Y*_*ij*_ refers to the standardized result of *y*_*ij*_

(3) Weight determination. Mean-Squared Deviation Weight Decision method is used to determine the weight of each index. The formulas are as follows equations:

The mean of random variable *G*_*j*_:

$$E(G_{j})=\frac{1}{n}\sum_{i=1}^{n}Y_{ij}$$ (5)

The mean square deviation of random variable *G*_*j*_:

$$F(G_{j})=\sqrt{\sum_{i=1}^{n}{\left(y_{ij}-E(G_{j})\right)}^{2}}$$ (6)

The weight of random variable *G*_*j*_:

$$W(G_{j})=F(G_{j})/\sum_{j=1}^{m}F(G_{j})$$ (7)

(4) Calculation of *K*. The value of *K* can be calculated by the weighted sum method below.


$$K_{i}=\sum_{j=1}^{m}\left(Y_{ij}*W(G_{j})\right)$$ (8)

Where *K*_*i*_ is the *i-th* city’s “attractive inertia exponent”; *W*(*G*_*j*_) is the weighted value of the *j-th* factor (index) that impacts the “attractive inertia exponent”; *Y*_*ij*_ is the standardized result of *y*_*ij*_.

#### Amending the method of measuring urban quality

In the existing literature, urban quality is generally represented by a single indicator [65], such as GDP, population size, gross industrial output value or fiscal revenue. However, a city is a complex entity composed of population, capital, technology, information, etc. Thus, the measurement of urban quality should take into account all the aspects mentioned above. Therefore, Multiple Attribute Decision Making (MADM) method is used to calculate urban quality. Table 1 above shows the index system for measuring urban quality.

The data pre-processing of this part is as same as that of the indexes of the “attractive inertia exponent” mentioned above. Eq (9) is the computational model of urban quality, which is also similar to that of “attractive inertia exponent”. Here, we multiply 100 for the formula of “attractive inertia exponent” in order to make the calculated results more reasonable and comparable to the others.


$$Q_{i}=100*\sum_{j=1}^{m}\left(Y_{ij}*W(G_{j})\right)$$ (9)

Where *Q*_*i*_ is the *i-th* city’s urban quality. *W*(*G*_*j*_) is the weighted value of the *j-th* factor (index) that impacts the “urban quality”; *Y*_*ij*_ is the standardized result of *y*_*ij*_.

#### Amending the method of measuring distance

This paper tries to combine the cost and time of inter-city transportation to define the comprehensive distance and replace the traditional distance by this comprehensive distance. In the Eq (10), this paper considers two main transportation modes of railway and highway because they are the two of the most common means connecting cities in Hunan Province and neighboring provinces. Considering the difference of the frequency of these traffic modes, the weight value of λ of the railway and highway is relatively 0.55 and 0.45.


$$d=\sum_{m=1}^{n}\lambda_{ij-m}c_{ij-m}T_{ij-m}$$ (10)

Where d is the comprehensive distance between city *i* and city *j*; *λ*_*ij−m*_ is the weight of the *m-th* mean of transportation from city *i* to city *j*; *c*_*ij−m*_ is the cost of the *m-th* mean of transportation from city *i* to city *j*; *T*_*ij−m*_ is the time of the *m-th* mean of transportation from city *i* to city *j*.

### Cluster analysis

As one of the popular classification methods, cluster analysis is deployed due to the advantage of its fast and straightforward characteristics. In this study, traditional provincial DEZ that relies on economic data purely is set as control group, whereas the proposed provincial DEZ derived from the new proposed framework in this research is viewed as experimental group. Both of these groups adopted the same clustering method for provincial DEZ but with different data.

#### Experimental group: Cluster analysis for provincial DEZ based on the matrix of urban gravity

According to the analytical framework for provincial DEZ, the cluster analysis is conducted based on the matrix of urban gravity, which is calculated by the modified gravity model mentioned above.

In order to make the cluster categories distinguishable, this study conducts two iterations of clustering. The first round of clustering targeted for the whole cities in Hunan Province is conducted firstly to identify the cities that should participate in the process of DEZ. After excluding the cities with smaller intra-province interaction (i.e., Chenzhou), we conduct the second clustering with the rest cities.

#### Control group: Cluster analysis for provincial DEZ based on the GDP economic data

The cluster analysis for DEZ in the control group is based on GDP economic data, which is viewed as one of the traditional methods of provincial DEZ. Two rounds of clustering are also made to make the categories more distinguishable. However, it should be noted that all of the cities, including Chenzhou, are involved in this cluster analysis because traditional methods don`t consider the influence of neighboring provinces.

## Findings and discussions

### Temporal changes of the city components in Each EZ

According to the external and internal force analysis, it should be noted that Chenzhou, which belongs to Hunan Province in terms of administrative management, was always excluded from the Hunan Province`s DEZ from 2002 to 2013 year, because its internal force was less than its external force (Table 2). Thus, from 2002 to 2013 year, there are only 13 cities that participate in Hunan Province`s DEZ, and Chenzhou can be named as Centrifugal City, which means to be a single and special part that does not involve in Hunan province`s DEZ but belongs to Hunan Province administratively.

**Table 2:** The external and internal forces of the border city in Hunan Province.

City	2002		2006		2009		2013	
	External force	Internal force	External force	Internal force	External force	Internal force	External force	Internal force
Huaihua	-1.59	9.21	-1.29	10.44	-1.03	6.80	-1.50	5.71
Changsha	-12.97	219.25	-12.60	218.95	-12.40	211.26	-19.19	219.45
Jishou	-0.60	31.72	-0.61	33.95	-0.40	18.02	-0.53	18.42
Shaoyang	-3.64	31.25	-2.34	20.78	-2.31	18.39	-1.95	16.99
Yongzhou	-8.09	29.65	-10.46	33.66	-4.48	13.94	-6.34	24.17
Yueyang	-5.40	76.68	-4.19	66.32	-3.28	59.55	-3.68	76.63
Zhangjiajie	-0.25	60.81	-0.20	70.57	-0.25	35.76	-0.15	23.78
Zhuzhou	-19.34	244.95	-22.22	252.96	-0.33	172.53	-0.31	194.66
Changde	-6.76	45.84	-6.11	66.75	-2.46	25.99	-2.46	19.39
**Chenzhou**	**-14.52**	**4.73**	**-16.78**	**6.16**	**-11.11**	**4.53**	**-10.29**	**4.02**

Based on the matrix of urban gravity (Table 3), we conduct a cluster analysis. It should note that the diagonal of the matrix in Table 3 refers to the urban gravity that the city acts on itself, this gravity is much larger than that from any of other cities, so it was set as 10000 according to its actual meaning. Here, we just present the matrix of urban gravity in 2002 due to the limited space.

**Table 3:** The urban gravity matrix of cities in Hunan Province in the 2002 year.

	Changsha	Yueyang	Xiangtan	Yiyang	Zhuzhou	Hengyang	Loudi	Changde	Huaihua	Zhangjiajie	Jishou	Yongzhou	Shaoyang
Changsha	10000	290.42	489.02	254.21	855.74	159.39	114.63	134.24	12.18	15.47	6.94	28.08	43.60
Yueyang	140.33	10000	27.23	18.27	75.31	24.15	15.37	22.07	3.79	3.92	2.16	7.24	8.76
Xiangtan	350.58	40.40	10000	26.44	2132.29	81.21	154.39	21.44	4.83	3.19	4.59	10.40	25.19
Yiyang	127.57	18.97	18.51	10000	26.37	19.99	65.81	124.11	2.45	6.11	2.54	5.34	19.90
Zhuzhou	543.23	98.94	1888.09	33.36	10000	115.14	89.84	29.00	5.93	4.09	2.46	15.31	25.91
Hengyang	82.40	25.84	58.56	20.59	93.77	10000	25.49	12.86	4.86	2.55	2.62	30.40	15.20
Loudi	52.13	14.46	97.93	59.63	64.36	22.42	10000	29.61	9.37	1.92	2.76	16.96	77.34
Changde	60.19	20.48	13.41	110.89	20.49	11.16	29.19	10000	6.36	24.75	7.40	7.73	19.03
Huaihua	6.36	4.09	3.52	2.55	4.88	4.90	10.75	7.40	10000	9.45	44.05	3.46	7.88
Zhangjiajie	7.62	4.00	2.20	6.00	3.18	2.43	2.08	27.20	8.92	10000	40.42	1.55	3.58
Jishou	3.11	2.00	2.86	2.27	1.74	2.26	2.72	7.38	37.76	36.68	10000	1.22	3.90
Yongzhou	10.49	5.60	5.42	3.97	9.01	21.98	13.94	6.45	2.47	1.18	1.02	10000	99.37
Shaoyang	21.07	8.76	16.98	19.16	19.72	14.21	82.19	20.51	7.30	3.50	4.21	128.48	10000

**Fig 3:**
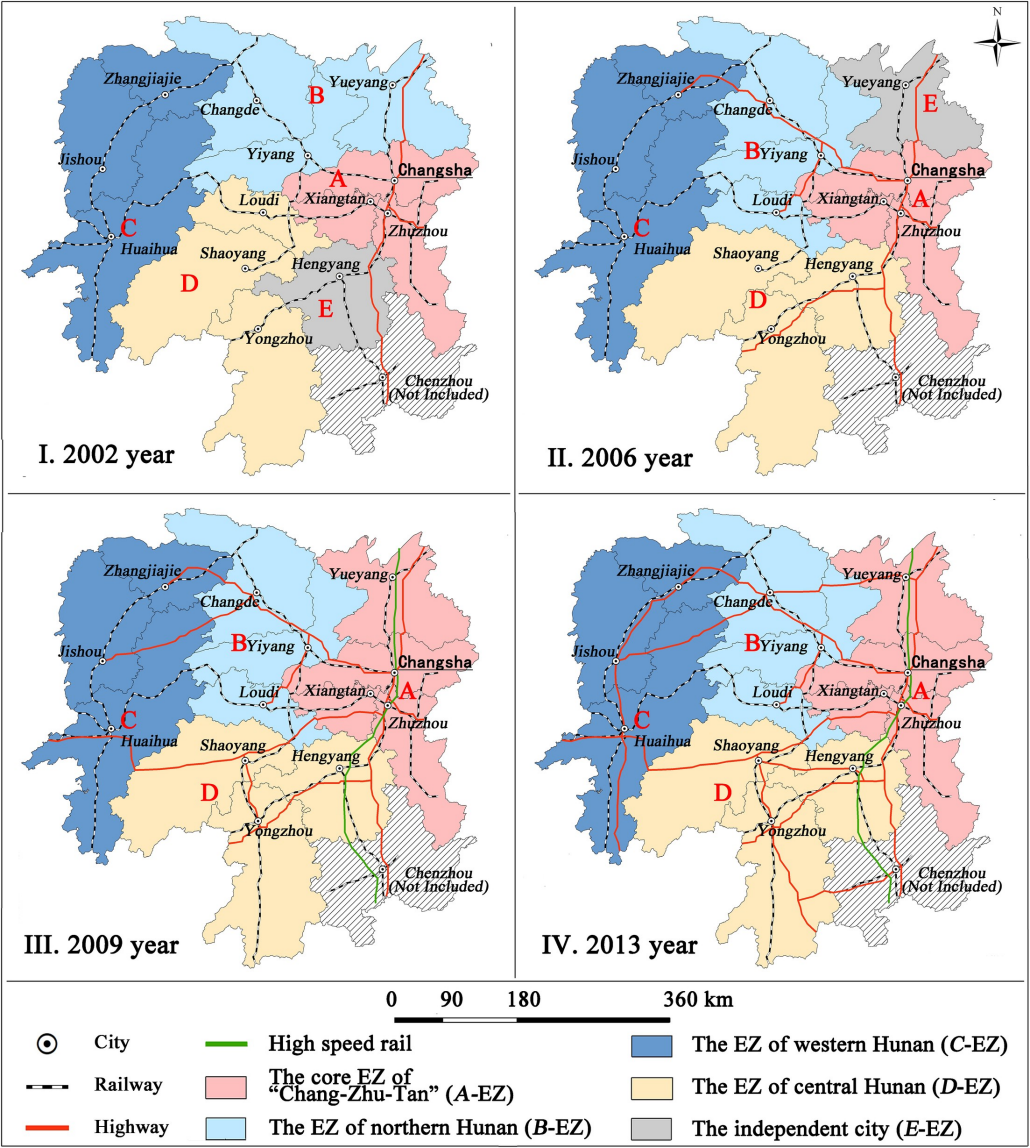
Temporal changes of the new proposed DEZ of Hunan Province from 2002 to 2013. (Reprinted from http://www.ngcc.cn/ngcc/ under a CC BY license, with permission from the *National Geomatics Center of China*, original copyright 2012).

Each cluster corresponds to one EZ respectively. Fig 3 shows the changes of the EZ components of cities at four years (Fig 3).

From 2002 to 2006 year, the constitutions of A-EZ (i.e., Chang-Zhu-Tan region) and C-EZ did not change. However, B-EZ changed greatly because Yueyang city detached itself from B-EZ and then came into being Independent City, which means to be a single and special part that does not belong to any EZ in Hunan Province although the city participates in the process of DEZ. Meanwhile, Loudi city, which belonged to D-EZ in 2002 year, joined in B-EZ. Another significant change is regarding the constitution of D-EZ. Loudi city separated itself from D-EZ, while its replacement was Hengyang city, which was an Independent City in 2002. In general, the components of cities in each EZ is unstable during the period. From 2006 to 2009, the constitutions of B-EZ, C-EZ, and D-EZ stay the same and just a few changes occurred in A-EZ. Yueyang city joined in A-EZ, rather than playing as an Independent City. That is, Yueyang has an increasing interaction with other cities in Hunan Province [66, 67]. Actually, there is no Independent City in Hunan Province anymore. From 2009 to 2013 year, no changes were observed. The components of cities in each EZ gradually has transformed into a stable status. The spatial patterns of EZs in 2009 and 2013 are appropriately consistent with the research by Xiong et al. [68].

In sum, the components of cities in EZs of Hunan Province begins with a great degree of fluctuation from 2002 to 2009 year, but followed by a phase of stability after 2009 year (Table 4). Although Chenzhou belongs to Hunan Province administratively, it was excluded from the DEZ of Hunan Province because its internal force is less than its external force. Here, it was concepted as Centrifugal City.

**Table 4:** The components of cities in each EZ from 2002 to 2013 year.

EZ (code and name)	Components of cities			
	2002	2006	2009	2013
**A:** the Core EZ of “Chang-Zhu-Tan”	Changsha, Zhuzhou, Xiangtan	Changsha, Zhuzhou, Xiangtan	Changsha, Zhuzhou, Yueyang, Xiangtan	Changsha, Zhuzhou, Yueyang, Xiangtan
**B:** the EZ of Northern Hunan	Changde, Yueyang, Yiyang	Changde, Loudi, Yiyang	Changde, Loudi, Yiyang	Changde, Loudi, Yiyang
**C:** the EZ of Western Hunan	Huaihua, Zhangjiajie, Jishou	Huaihua, Zhangjiajie, Jishou	Huaihua, Zhangjiajie, Jishou	Huaihua, Zhangjiajie, Jishou
**D:** the EZ of Central Hunan	Yongzhou, Loudi, Shaoyang	Yongzhou, Hengyang, Shaoyang	Yongzhou, Hengyang, Shaoyang	Yongzhou, Hengyang, Shaoyang
**E:** the Independent City	Hengyang	Yueyang	——	——
**Centrifugal City** (Excluded from DEZ)	Chenzhou	Chenzhou	Chenzhou	Chenzhou

### Comparisons between the traditional and new proposed DEZ schemes

**Fig 4:**
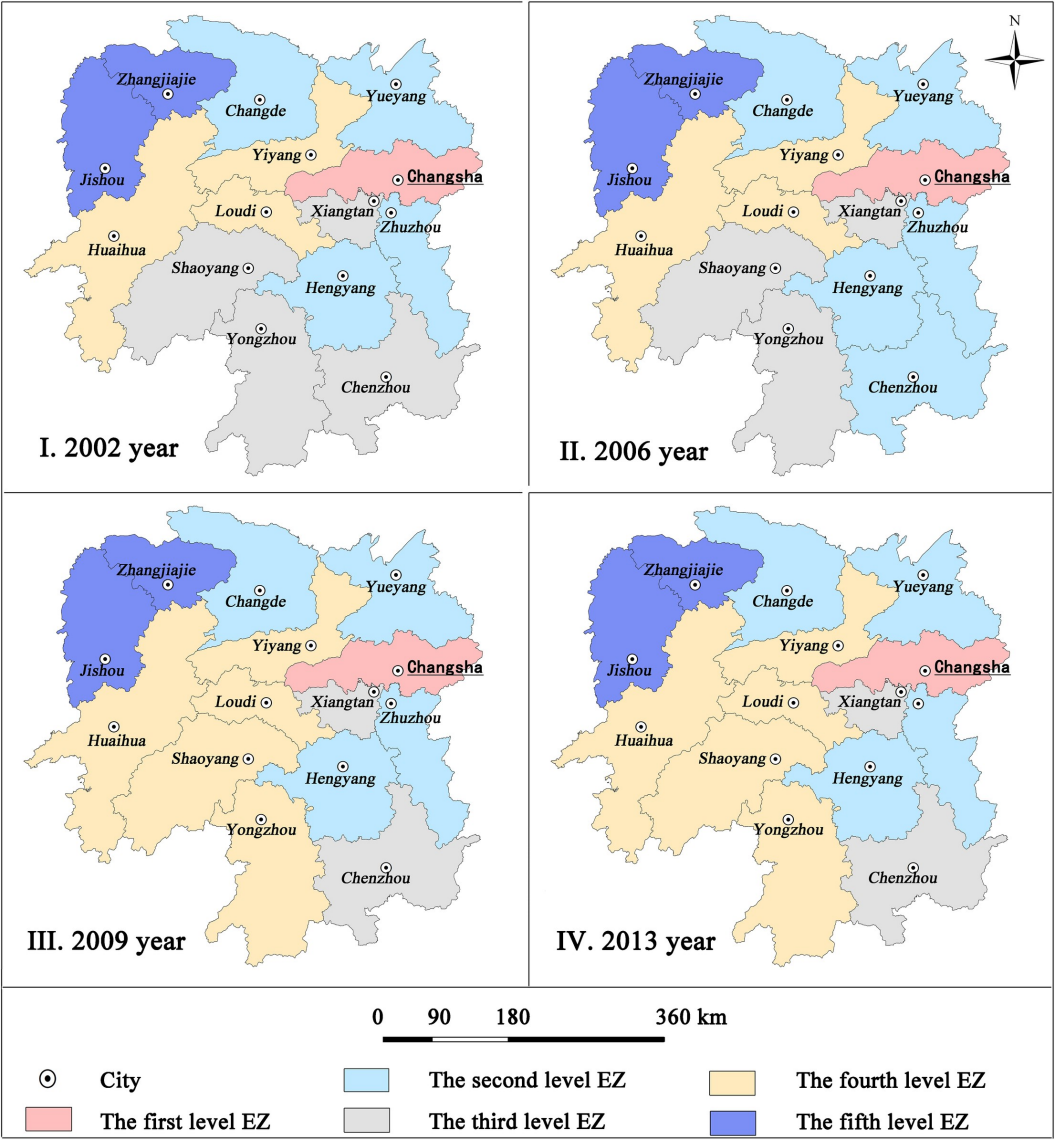
Temporal changes of traditional DEZ scheme of Hunan Province from 2002 to 2013. (Reprinted from http://www.ngcc.cn/ngcc/ under a CC BY license, with permission from the *National Geomatics Center of China*, original copyright 2012).

Fig 4 shows the evolution of DEZ and spatial distribution of EZ, which is derived from the cluster analysis based on GDP economic data purely. Fig 3 illustrates the evolution of DEZ and spatial distribution of EZ, which is derived from the cluster analysis based on the matrix of urban gravity (Table 3). The comparison between these figures shows that DEZ based on GDP economic is significantly different from that based on the matrix of urban gravity.

The traditional method of provincial DEZ often tends to use GDP economic data, because it is easy to access. Additionally, GDP is also one of the major indexes that can represent the economic development of a region. Based on GDP economic data purely, the traditional method applies the cluster analysis and regards each cluster as EZ respectively. It is obvious that EZs in Fig 4, which shows the evolution of DEZ of Hunan Province distribute with a characteristic of geographic discontinuity at each time point. In 2002, Changsha, which was clustered into the first level EZ, divided the second level EZ into two parts. As a result, cities in the second level EZ are not adjacent to each other (i.e., discontinuous geographically). The same separation was also observed in the third level EZ. The EZ was composed of four cities in total and was divided by Hengyang. Geographic discontinuity further causes difficulty in regional economic collaborations among cities in a discontinuous EZ, because two adjacent cities tend to have more economic contact with each other than two non-adjacent ones. For example, Zhuzhou, which belongs to the second level EZ, would interact easily and frequently with Changsha, which belongs to the first level EZ, but not with Yueyang, which belongs to the second level EZ. Thus, such a traditional scheme of DEZ based on GDP economic data purely can`t fully represent the spatial structure of EZ in that it does not consider the geographical property of EZ and often shows a feature of geographic discontinuity [33].

Comparatively speaking, Fig 3 shows the evolution of the DEZ of Hunan Province based on the matrix of urban gravity. It has the advantage of geographic continuity regarding the spatial distribution of EZs. All EZs at each time point are continuous geographically. This characteristic shows that the analytical framework for provincial DEZ based on the matrix of urban gravity is better suited for DEZ theoretically and practically.

### Impact of traffic conditions on DEZ

During the change of the components of cities in each EZ (Table 4), the traffic condition has been playing a very important role. The development of transportation infrastructure has a great impact on the interaction between the cities, which belong to different EZs. Once the traffic condition between these cities is improved, they tend to interact with each other more frequently. Thus, one of these cities will merge into another EZ after escaping from its previous EZ. As a result, the city components of EZs will change.

In Hunan Province, highway and high-speed rail completely replaced national road, one type of road with relatively narrow width and low safety in China, and have covered most of Hunan Province in 2013. The highway and high-speed rail have great advantages over the national road due to their speed and safety. All kinds of communication of all kinds, including economic and cultural contacts, passenger and freight transport, become more and more convenient and frequent. As a result, this change of mutual links significantly affects the city components of EZs in Hunan Province.

According to Fig 3, a highway was constructed between Yongzhou and Hengyang, as well as between Loudi and Yiyang from 2002 to 2009. In the meanwhile, the city components of B-EZ and D-EZ also changed greatly. Obviously, this change above is not a coincidence. The highway has played a significant role because the highway between Yongzhou and Hengyang strengthens the link between these two cities and it is helpful for Hengyang to join in D-EZ. Besides, another highway between Loudi and Yiyang helped Loudi detach from D-EZ and turn to B-EZ in that this highway indeed has enhanced the transport between Loudi and Yiyang. Based on these pieces of evidences, it is concluded that the highway has been playing an important role in changing the spatial distribution of EZs in Hunan.

From 2009 to 2013, a high-speed rail was built through the north of Hunan Province to its south, while Yueyang joined in A-EZ instead of being an Independent City. Based on the same thought, high-speed rail also involved in the changes of the spatial structure of economic zones because high-speed rail reduced transport time dramatically between Yueyang and Changsha [69]. This convenience greatly promoted the interaction between them and helped Yueyang integrate itself into the A-EZ.

In sum, the construction of transport infrastructure and the change of the city components in each EZ, highway and high-speed rail indeed have been playing an important role in changing the geographical distribution of EZ in Hunan Province.

### Impact of cross-provincial regional integration on DEZ

**Fig 5:**
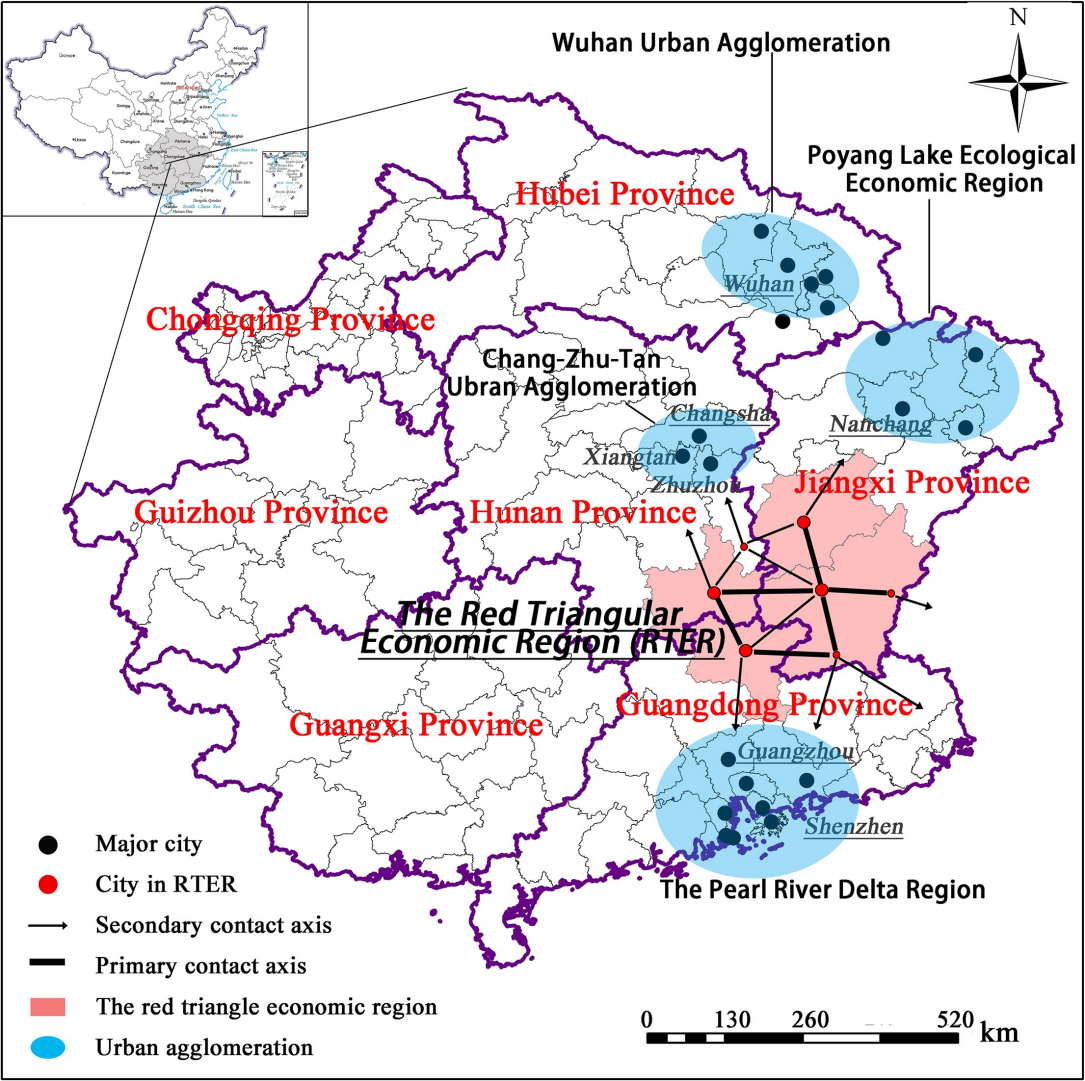
Cross-provincial regional integration and economic contact in RTER. (Reprinted from http://www.ngcc.cn/ngcc/ under a CC BY license, with permission from the *National Geomatics Center of China*, original copyright 2012).

Chenzhou is far away from the core area of Hunan Province (Fig 5). The situation is similar to that of Ganzhou (a boundary city of Jiangxi Province) and Shaoguan (a boundary city of Guangdong Province). Because of the provincial government`s less attention to or investment in these boundary cities, they tend to fell behind the cities in the core zone [68]. However, these border cities also try to change their current situation by cooperating with each other. They establish certain collaboration organizations to strengthen the interaction among themselves. For instance, Ganzhou, Chenzhou, and Shaoguan set up the regional organization of *Red Triangular Economic Region* (RTER) together in 2004 based on their advantages of tourism [70]. In 2005, these cities further formulate the *Regional Cooperation Framework Agreement*, which aimed at propelling regional cooperation in all fields of economic development, not only in the tourism industry. Besides, in 2014, Chenzhou and Shaoguan proposed to build up an *Open Cooperation Experimental Zone*, which means that these two cities will collectively plan and build infrastructure and uniformly distribute major industries. These plans have strengthened the connections among these cities and promoted cross-provincial regional integration. From another perspective, Chenzhou, despite under the administration of Hunan Province, has fewer ties with cities in Hunan Province.

According to Table 2, Chenzhou is the only city in Hunan Province that did not participate in the process of DEZ in 2002, 2006, 2009, and 2013. In each of these years, its external force is much larger than its internal force. For example, the value of its external force (14.52) vs. the internal force (4.733) in 2002. It demonstrates that Chenzhou has been excluded from the process of DEZ in Hunan Province because of its fewer ties with the city in Hunan Province.

## Conclusions

During the process of regional development, regions usually endow distinctive advantages as well as exposing different disadvantages. It requires the local governments to achieve the sustainable development at regional level by utilizing the comparative advantages effectively. Therefore, DEZ which highlights the regional collaborations based on the distinctive spatial interactions, is applicable to contribute to the goal of sustainable development. In this paper, an analytical framework of provincial DEZ, which is based on the improved gravity model and clustering approaches, is proposed and then applied to a case study of Hunan Province, China.

The results are summarized. First, it reveals that Chenzhou city in the south of Hunan Province is always excluded from the DEZ due to its larger external gravity from other cities in neighboring provinces. It demonstrates that border cities could have a strong connection with the surrounding cities in neighboring provinces. Second, the city components of EZs in Hunan Province changed greatly from 2002 to 2013, particularly featured with a great degree of fluctuation from 2002 to 2009, followed by a phase of stability after 2009. Such changes of city components are majorly contributed to the transportation development, especially the highway construction throughout the Hunan Province. Third, cross-provincial regional integration among local municipal governments, which usually share similar social and cultural background context, has a significant impact on the changes of city components of EZ.

This study may also contribute to the current researches in several aspects. Comparing with the traditional DEZ method that purely uses GDP economic data to conduct cluster analysis, the framework in this paper that is based on the matrix of urban gravity can better reveal the geographic continuity feature of EZ. Such continuity of the cities in EZ is important for the formation of EZ because neighboring cities may have high possibility of economic, cultural, and social interactions. Second, our framework of the provincial DEZ also takes the cross-provincial interaction for border cities into account. By comparing the internal force and external force that the border city faces, this study demonstrates that it is necessary to consider the spatial interaction between border cities and its neighboring cities in other provinces when forming the provincial DEZ. Furthermore, the proposed framework of provincial DEZ provides a quantitative method to evaluate the potentials of regional collaboration among cities within a provincial space. The key idea of regional collaboration of provincial DEZ would greatly help to find a solution to achieve the sustainable development of a province, thereby contributing to the regional planning, government management, and regional policy in practice.

The limitation of this research is that the index system for calculating the urban quality and the “attractive inertia exponent” may not fully comprehensive because of the difficulty in data availability of possible indicators. For example, population, transportation, and economic trade flows among cities are more applicable to present the interactions. However, the data on the flow information is not available. Furthermore, it is recommended to conduct the provincial DEZ at a smaller scale (i.e., county level), which may present more rich information on spatial interaction. Third, the internetization and informatization have been demonstrated to contribute to the regional development recently, and hence, future studies may consider the effects of these two factors on provincial DEZ.

## Supporting information

S1 Data (XLSX)

## References

[1] Shertzer A, Twinam T, Walsh RP. Zoning and the economic geography of cities. Journal of Urban Economics. 2018; 105: 20–39. 10.1016/j.jue.2018.01.006

[2] Liu B. Research on the economic regionalization of China. China Soft Science. 2009; 2: 81–90 (in Chinese). 10.3969/j.issn.1002-9753.2009.02.010

[3] World Bank. World Development Indicators. 2012. http://data.worldbank.org/ data-catalog/world-development-indicators.

[4] Wei YD, Fan CC. Regional inequality in China: A case study of Jiangsu Province. Professional Geographer. 2000; 52: 455–469. 10.1111/0033-0124.00238

[5] World Bank. World development report 2006: equity and development. Oxford University Press: New York, USA, 2005.

[6] Yeh AGO. China’s Pan-Pearl River Delta: regional cooperation and development. University of Washington Press: Seattle, USA, 2011.

[7] Wedeman A. The double paradox of rapid growth and rising corruption in China. Cornell University Press: New York, NY, USA, 2012.

[8] Li H, Zhou LA. Political turnover and economic performance: the incentive role of personnel control in China. Journal of public economics.2005; 89: 1743–1762. 10.1016/j.jpubeco.2004.06.009

[9] Yang W, Liang J. Exploration of China’s top ten economic zones. Economic Geography. 1992; 3:14–20 (in Chinese).

[10] Wang X, Fan G. Analysis on the regional disparity in China and the influential factors. Economic Research Journal. 2004; 2(4): 34–44.

[11] He S, Bayrak MM, Lin H. A comparative analysis of multi-scalar regional inequality in China. Geoforum. 2017; 78:1–11. 10.1016/j.geoforum.2016.10.021

[12] Duan Q, Mao J. Economic regionalization in provincial cope based on the gravity model and 0–1 programming model. Economic Geography. 2011; 31:1239–1245 (in Chinese). 10.15957/j.cnki.jjdl.2011.08.002

[13] Zhu D, Lu L. Spatial patterns of city in Anhui Province based on gravity model. Scientia Geographica Sinica. 2011; 5: 551–556 (in Chinese). 10.13249/j.cnki.sgs.2011.05.006

[14] Geng C, Liu Z. Research on spatial patterns of cities in Shandong Province based on gravity model. Journal of Shandong Jianzhu University. 2012; 27: 302–306 (in Chinese). 10.3969/j.issn.1673-7644.2012.03.011

[15] Emilian MD, Edith MD. Theories regarding the role of the growth poles in the economic integration. Procedia Economics and Finance. 2014; 8: 262–267. 10.1016/S2212-5671y

[16] Jiang L, Yang K. Research on the standard method of economic regionalization in China. Hubei Social Sciences. 2007; 6: 72–76 (in Chinese). 10.3969/j.issn.1003-8477.2007.06.022

[17] Chen Y. et al. *Spatial pattern and the development strategy of the county economic under the provincial multi gravity*: *Take Hakka neighborhoods of Guangdong*, *Fujian and Jiangxi as examples*. *Economic Geography*. 2018; 38(1): 46–51+141. 10.15957/j.cnki.jjdl.2018.01.006

[18] Liang Y, Zhou Z, Li X. Dynamic of regional planning and sustainable development in the Pearl River Delta, China. Sustainability. 2019; 11(21): 6074. 10.3390/su11216074

[19] Liu Y, Wang F, An N. The duality of political geography in China: Integration and challenges. Geopolitics. 2020; 25(4), 968–988. 10.1080/14650045.2018.1465042

[20] Fang C, Liu H, Luo K, Yu X. Process and proposal for comprehensive regionalization of Chinese human geography. Journal of Geographical Sciences. 2017; 27(10): 1155–1168. 10.1007/s11442-017-1428-y

[21] Habeeb NJ, Weli ST. Relationship of smart cities and smart tourism: an overview. High Tech and Innovation Journal. 2020; 1(4): 194–202. 10.28991/HIJ-2020-01-04-07

[22] Ye XY, Michael CC. Exploratory space-time analysis of local economic development. Applied Geography. 2011; 31: 1049–1058. 10.1016/j.apgeog.2011.02.003

[23] Yang S. National land consolidation and economic regionalization. Economic Geography. 1982; 2: 252–255 (in Chinese).

[24] Yang K, Jiang L. The transition of China’s economic regionalization and the forward topics. Chinese Public Administration. 2010; 5: 79–82. (in Chinese)

[25] Wei YD, Ye X. Beyond convergence: space, scale, and regional inequality in China. Tijdschrift voor Economische en Sociale Geografie. 2009; 100: 59–80. 10.1111/j.1467-9663.2009.00507.x

[26] Wei Y. Regional development in China: Transitional institutions, embedded globalization, and hybrid economies. Eurasian Geography and Economics. 2007; 48(1): 16–36. 10.2747/1538-7216.48.1.16

[27] Zhang H, Zhang S, Liu Z. Evolution and influencing factors of China’s rural population distribution patterns since 1990. Plos ONE. 2020; 15(5): e0233637. doi: 10.1371/journal.pone.0233637 32470034 PMC7259518

[28] Li H, et al. Research on the evolution of regional economic pattern and the optimization of spatial strategic structure in Hunan Province. Economic Geography. 2020; 40(11): 39–46+85 (in Chinese). 10.15957/j.cnki.jjdl.2020.11.005

[29] Guo Z. Economic region and division of economic region. Price Press: Beijing, China, 1998 (in Chinese).

[30] Gao X, Wang Y, Mai S. Research on economic regionalization in the development of regional economy: Based on the thinking of conflict between economic divisions and administrative divisions. Guizhou Social Sciences. 2010; 11: 72–76 (in Chinese). 10.3969/j.issn.1002-6924.2010.11.015

[31] Yeh AGO, Xu J, Liu K. China’s post-reform urbanization: retrospect, policies and trends. The report on urbanization and emerging population issues-5. Human settlements group, IIED, population and development branch, UNFPA, 2011. http://www.iied.org/pubs/display.php?o=10593IIED.

[32] Wu Y, Shan L, Zheng S, Lai SK, Xia B. Regional planning reconfiguration in China based on inclusiveness: Examining development and control orientation. Journal of Urban Planning and Development. 2020; 146(3): 05020012. 10.1061/(ASCE)UP.1943-5444.0000578

[33] Fan J. The scientific foundation of major function-oriented zoning in China. Acta Geographica Sinica. 2007; (4): 339–350 (in Chinese). 10.3321/j.issn:0375-5444.2007.04.001

[34] Rutt S. The Soviet concept of the territorial-production complex and regional development. The Town Planning Review. 1986; 57(4): 425–439. 10.2307/40113582

[35] Jacob OM, Israel P. The spatial organization of rural services: An operational model for regional development planning. Applied Geography. 1988; 8: 65–79. 10.1016/0143-6228(88)90006-9

[36] Patrizia T, Steve C. Multi-criteria, multi-objective and uncertainty analysis for agro-energy spatial modelling. Applied Geography. 2012; 32: 724–736. 10.1016/j.apgeog.2011.08.013

[37] Kenneth PJ, John RK. Redefinition of the BEA economic areas. Survey of Current Business. 2004; 84: 45–52.

[38] Self PJO. Regional planning in Britain: Analysis and evaluation. Regional Studies.1967, 1(1): 3–10. 10.1080/09595236700185021

[39] Raymond C. The regional model: A regional-national model for French planning. Regional Science and Urban Economics. 1979; 9: 117–139. 10.1016/0166-0462(79)90009-7

[40] Bernard C. Indicative planning in France. Journal of Comparative Economics. 1990; 14: 607–620. 10.1016/0147-5967(90)90042-8

[41] New York Government. http://regionalcouncils.ny.gov/, 2011

[42] Alberta Economic Development Authority. http://eae.alberta.ca/economic-development/regional-development/redas.aspx, 1999.

[43] Richardson H. Regional and Urban Economics. Pitman Publishing: London, UK, 1973.

[44] Field BG, Macgregor BD. Forecasting techniques for urban and regional planning. Hutchinson: London, UK, 1987.

[45] Anderson JE. A theoretical foundation for the gravity equation. American economic review. 1979; 69: 106–160. 10.2307/1802501

[46] Tinbergen J. Shaping the world economy: suggestions for an international economic policy. Twentieth-Century Fund: New York, NY, USA, 1962.

[47] Anderson JE, Wincoop EV. Gravity with gravitas: A solution to the border puzzle. American economic review. 2003; 93: 170–192. 10.1257/000282803321455214

[48] Stewart JQ. The development of social physics. American Journal of Physics. 1950; 18: 239–253. 10.1119/1.1932559

[49] Matsumoto H. International urban systems and air passenger and cargo flows: Some calculations. Journal of Air Transport Management. 2004; 10: 214–249. 10.1016/j.jairtraman.2004.02.003

[50] Bergstrand JH. The generalized gravity equation, monopolistic competition, and the factor-proportions theory in international trade. The review of economics and statistics. 1989; 71: 143–153. 10.2307/1928061

[51] Bergstrand JH, Egger P. A knowledge and physical-capital model of international trade flows, foreign direct investment and multinational enterprises. Journal of International Economics. 2007; 73: 278–308. 10.1016/j.jinteco.2007.03.004

[52] Okeke FO., Eziyi IO, Udeh CA, Ezema EC. City as Habitat: Assembling the fragile city. Civil engineering Journal. 2020; 6(6): 1143–1154. 10.28991/cej-2020-03091536

[53] Qerimi D, Dimitrieska C, Vasilevska S, Rrecaj AA. Modeling of the solar thermal energy use in urban areas. Civil Engineering Journal. 2020; 6(7): 1349–1367. 10.28991/cej-2020-03091553

[54] Thompson CA, Saxberg K, Lega J, Tong D, Brown HE. A cumulative gravity model for inter-urban spatial interaction at different scales. Journal of Transport Geography. 2019; 79: 102461. 10.1016/j.jtrangeo.2019.102461

[55] Melkumian AV. A gravity model of legal migration into the United States. Journal of Economic Development & Business Policy. 2009; 2: 1–18.

[56] Head K, Ries J. FDI as an outcome of the market for corporate control: Theory and evidence. Journal of International Economics. 2008; 74: 2–20. doi: 10.1016/j.jinteco.2007.04.004

[57] Kabir M, Salim R, Al-Mawali N. The gravity model and trade flows: Recent developments in econometric modeling and empirical evidence. Economic analysis and policy. 2017; 56: 60–71. 10.1016/j.eap.2017.08.005

[58] Okubo T. The border effect in the Japanese market: A gravity model analysis. Journal of the Japanese and International Economies, 2004; 18: 1–11. 10.1016/s0889-1583(03)00047-9

[59] Keum K. Tourism flows and trade theory: A panel data analysis with the gravity model. The Annals of Regional Science volume. 2010; 44: 541–557. 10.1007/s00168-008-0275-2

[60] Krings G, Calabrese F, Ratti C. Urban gravity: A model for inter-city telecommunication flows. Journal of Statistical Mechanics: Theory and Experiment. 2009; 7: L07003. 10.1088/1742-5468/2009/07/l07003

[61] Lenormand M, Huet S, Gargiulo F, Deffuant G. A universal model of commuting networks. PloS ONE. 2012; 7(10): e45985. doi: 10.1371/journal.pone.0045985 23049691 PMC3462197

[62] Wu C, Smith D, Wang M. Simulating the urban spatial structure with spatial interaction: A case study of urban polycentricity under different scenarios. Computers, Environment and Urban Systems. 2021; 89, 101677. 10.1016/j.compenvurbsys.2021.101677

[63] Smith DA, Timberlake M. Conceptualizing and mapping the structure of the world system’s city system. Urban Studies. 1995; 32: 287–302. 10.1080/00420989550013086

[64] Liu X, Taylor PJ. Research note-a robustness assessment of global city network connectivity rankings. Urban Geography. 2011; 32: 1227–1237. 10.2747/0272-3638.32.8.1227

[65] Wang X, Wu D, Wang H. An attempt to calculate economic links between cities. *Urban Studies*. 2006; 3: 55–59 (in Chinese). 10.3969/j.issn.1006-3862.2006.03.011

[66] Zheng Y, Xu W, Dai L. Urban growth in a post‐2000 central Chinese urban agglomeration: Case study of the Changzhutan region. Growth and Change. 2020; 51(1): 464–87. 10.1111/grow.12360

[67] Quan B, Xiao Z, Römkens M, Bai Y, Lei S. Spatiotemporal urban land use changes in the Changzhutan region of Hunan Province in China. Journal of Geographic Information System. 2013; 5(2): 136–147. 10.4236/jgis.2013.52014

[68] Xiong Y, Wang K, Wen X. Disparity and temporal-spatial structure characteristics of regional economic development in Hunan Province. Resources and Environment in the Yangtze Basin. 2008; 17(1): 22–29 (in Chinese). 10.3969/j.issn.1004-8227.2008.01.005

[69] Zhang X. Analysis of economic space change in Hunan Province. Contemporary Economics. 2018; 2: 92–93 (in Chinese). 10.3969/j.issn.1007-9378.2018.02.039

[70] Xiao H, Gu R, Huang J. On the combined developing mode of the Red Triangle Region’s Tourism resources. World Regional Study. 2010; 19(3): 121–127 (in Chinese). 10.3969/j.issn.1004-9479.2010.03.016

